# Quantifying skeletal muscle volume and shape in humans using MRI: A systematic review of validity and reliability

**DOI:** 10.1371/journal.pone.0207847

**Published:** 2018-11-29

**Authors:** Christelle Pons, Bhushan Borotikar, Marc Garetier, Valérie Burdin, Douraied Ben Salem, Mathieu Lempereur, Sylvain Brochard

**Affiliations:** 1 Pediatric rehabilitation department, Fondation ILDYS, Brest, France; 2 Laboratoire de Traitement de l’Information Médicale, INSERM, Brest, France; 3 Radiology department, hôpital d'Instruction des Armées Clermont-Tonnerre, Brest, France; 4 IMT Atlantique, Brest, France; 5 Université de Bretagne Occidentale, Brest, France; 6 Radiology department, CHRU de Brest, Brest, France; 7 PMR department, CHRU de Brest, Hopital Morvan, Brest, France; Universite de Nantes, FRANCE

## Abstract

**Aims:**

The aim of this study was to report the metrological qualities of techniques currently used to quantify skeletal muscle volume and 3D shape in healthy and pathological muscles.

**Methods:**

A systematic review was conducted (Prospero CRD42018082708). PubMed, Web of Science, Cochrane and Scopus databases were searched using relevant keywords and inclusion/exclusion criteria. The quality of the articles was evaluated using a customized scale.

**Results:**

Thirty articles were included, 6 of which included pathological muscles. Most evaluated lower limb muscles. Partially or completely automatic and manual techniques were assessed in 10 and 24 articles, respectively. Manual slice-by-slice segmentation reliability was good-to-excellent (n = 8 articles) and validity against dissection was moderate to good(n = 1). Manual slice-by-slice segmentation was used as a gold-standard method in the other articles. Reduction of the number of manually segmented slices (n = 6) provided good to excellent validity if a sufficient number of appropriate slices was chosen. Segmentation on one slice (n = 11) increased volume errors. The Deformation of a Parametric Specific Object (DPSO) method (n = 5) decreased the number of manually-segmented slices required for any chosen level of error. Other automatic techniques combined with different statistical shape or atlas/images-based methods (n = 4) had good validity. Some particularities were highlighted for specific muscles. Except for manual slice by slice segmentation, reliability has rarely been reported.

**Conclusions:**

The results of this systematic review help the choice of appropriate segmentation techniques, according to the purpose of the measurement. In healthy populations, techniques that greatly simplified the process of manual segmentation yielded greater errors in volume and shape estimations. Reduction of the number of manually segmented slices was possible with appropriately chosen segmented slices or with DPSO. Other automatic techniques showed promise, but data were insufficient for their validation. More data on the metrological quality of techniques used in the cases of muscle pathology are required.

## Introduction

The volume and shape of a muscle are strongly related to its function [1–4]. Structural differences between muscles, which result from different muscle fibre architecture, are good predictors of force generation capacity [1]. Physiological cross-sectional area is the major determinant of joint torque [1]. Muscle volume, which is closely related to physiological cross sectional area, was shown to be strongly connected with joint torque in both healthy and pathological populations [2–5]. Changes in muscle volumes and shapes may be normal, such as hypertrophy after a strengthening program, or atrophy associated with ageing [6,7]. Changes can also be pathological due to neuromuscular disease or injury [5,8,9].

Assessment of muscle volume and shape is essential for both clinical practice and research. Measurement of muscle volume facilitates surveillance of neuromuscular disease progression [10,11] and the effects of treatments [12,13], as well as being useful for diagnostic purposes [14,15]. Muscle shapes can be used to distinguish between pathologies [16,17] and modelling individual muscles can be useful when planning surgery [18], evaluating changes over time [6,19] and in order to improve the understanding of particular symptoms or diseases [16,17,20–22].

Magnetic resonance imaging (MRI) is the gold-standard technique for the evaluation of muscle volumes and three-dimensional (3D) shapes, and is used as a reference to validate other imaging techniques for this purpose [23,24]. Many manual and automatic segmentation techniques have been developed for the estimation of muscle volumes and 3D shapes from MRI data [25–29]. However, despite the widespread use of these measurements in both clinical practice and research, to date neither their metrological qualities nor their feasibility for use in routine practice have been specifically reviewed.

Knowledge of the validity and reliability of measurement methods is essential when choosing a technique in order to ensure an accurate interpretation of the results [30,31]. Validity is the degree to which a technique measures what it is intended to measure, and the extent to which the values obtained are similar to the true values. Reliability is the extent to which a technique yields the same results over repeated trials in stable study subjects [31,32]. Techniques that are easy to use may lack validity or reliability whereas techniques that are valid and reliable are not always feasible for use in a research or clinical setting if they are too time-consuming. It may thus be necessary to compromise between (I) the metrological accuracy required and (II) practical considerations of usage.

The main aim of this systematic review was to report the validity and reliability of techniques used to estimate skeletal muscle volumes and 3D muscle shapes based on MRI data in healthy and pathological muscles in humans. The secondary aims were to determine the feasibility of those techniques and to provide recommendations for future research.

Our first hypothesis was that manual slice by slice segmentation would have good metrological properties but would need a large amount of time. The second was that while providing valid and reliable results, automatic segmentation techniques would require less time.

## Materials and methods

This systematic review adheres to the PRISMA guidelines. A PRISMA checklist was completed (S1 Table) and the review protocol was published in Prospero (CRD42018082708).

### Database search and selection process

Articles were identified through a comprehensive search of the following online bibliographic databases: PubMed, Web of Science, The Cochrane Library and Scopus. In order to ensure the search was exhaustive, the following Medical Subject Headings (MeSH) and keyword combinations were used (I) MRI, magnetic resonance imaging, (II) muscle, skeletal muscle, muscul* (III) (keywords relating to segmentation) volum*, cross sectional area, three dimension*, 3D, shape, segmentation, organ size and (IV) (keywords relative to metrological properties) reliability, reproducibility, repeatability, validity, accuracy, measur*, metrologic*, validation stud*. Search strings were formulated and tailored to the search syntax of each database to ensure a common search strategy (S1 Text). Neither publication year nor language limits were imposed. The last search was performed in January 2018.

Inclusion criteria were: I) studies in which the main aim was to describe and/or evaluate a method to determine skeletal muscle (or functional groups) volume and/or shape using MRI data, II) the study was on human subjects and III) the study included an evaluation of the metrological qualities of the method. Studies that evaluated head and neck muscles or that evaluated muscle groups that were not functionally grouped [33,34] and conference papers were excluded. Articles that compared ultrasonography (USG) with MRI to evaluate MRI validity were also excluded [35]. The references of the selected articles were screened to complete the review process. The titles, abstracts and whole texts of the articles identified by the search were independently evaluated by two examiners (CP and ML). Any disagreements were resolved by discussion between the two examiners.

### Quality assessment of selected studies

Since no standardized tools exist to determine the quality of articles in the field of radiology, a customized quality assessment scale was developed from other scales in the literature [36,37]. The aim of the scale was to assess both the intrinsic quality of each article (maximum score 30) and the metrological qualities of the method evaluated (maximum score 11). The total score was named the Q score and was out of 100. The first (quality) part of the scale was based on previously published quality checklists for systematic reviews as well as scales for the assessment of the quality of studies included in systematic reviews. Those scales included questions relating to study design and quality of the reporting of methodologies and results [38–40], for example “were the aims clearly stated” or “was the description of patient recruitment clear” (S2 Table). The second (metrological) part of the scale was based on published scales that were specifically designed for the evaluation of metrological studies in other fields than radiology [31,36,37,41,42]. It included questions such as “was concurrent validity evaluated?” or “Was the gold standard measure described?”. The grades for the questions ranged from 0 to 2. This scale was only used for the purposes of the present study. The quality rating was carried out independently by two examiners (CP and BB) and disagreements were resolved by consensus.

### Data extraction and analysis

Information regarding the samples included, muscles evaluated, magnetic field strengths and MRI protocols used were collected from each article. The technique evaluated, the reference technique used, operators and outcome measures (validity, reliability and feasibility) were also recorded (Table 1 and S3 Table). In this paper, validity refers to the concept of concurrent validity [31] and reliability refers to the correlations between different measurements within the same stable subject, as well as the measurement error [30,43]. To assess the validity and reliability of the results reported in each article, the following values were considered: standard error of the estimate (SEE) and root mean square error (RMSE), values > 10% = poor, 5–10% = moderate, 1–5% = good and < 1% = excellent. The same limits were used for the coefficient of variation. Mean differences, results > 5% = poor, 2–5% = moderate, 1–2% = good and < 1% = excellent. For mean distances, results with distances > 6 mm = poor, 3–6 mm = moderate, 1–3 mm = good and < 1 mm = excellent. Intraclass correlation coefficients (ICC) and r^2^ values from 0–0.49 = poor, 0.5–0.69 = moderate, 0.7–0.89 = good and > 0.9 = excellent [44]. The same limits were used for the Dice similarity index(DSI). DSI is the size of the overlap of the two segmentations divided by the total size of the two objects. If different statistical analyses were available in the same study, the worst results were primarily used for the classification. Although we acknowledge that there is no reference or reported recommendation for this categorization, it was used to provide clarity and to standardize the hierarchy of the results reported in the selected articles. The results for validity and reliability were also extracted as they were reported in each original article (S4 Table). When similar evaluations were carried out, for example a bilateral psoas evaluation in a healthy subject using the same technique for each side [45], only the poorest values of validity or reliability were reported. Technique feasibility was determined as the time required for manual segmentation to be carried out or from the time needed to run automatic techniques.

**Table 1:** Description of the segmentation techniques and methodology of the articles included.

	Muscles evaluated	reference technique	technique (methodology and volume/shape calculation)	optimization of the acquisition for targeted error	operators		Outcome measures		Statistical analysis
					number, qualification and experience	reliability study design	volume/ 3D shape		
**Albracht 2008 [52]**	GM, GL, SO	slice by slice manual segmentation, volume using 3D shape	single slice manual segmentation (CSAmax), muscle length (ML) obtained using full muscle reconstruction and shape factor (p)determined in a group with untrained and trained persons, volume: p* CSAmax* ML	-	-	-	volume	concurrent validity	volume RMSE
** **	* *								
**Amabile 2017 [53]**	QL, ES, GlMa, GlMe, GlMi, AddOP, VLI, VM, TFL, RF, Gra, Sar, BFS, BLF, SM, ST, grouped in spine extensors/flexors, hip extensors/flexors, knee extensors/flexors, both sides	3D reconstruction, segmentation using parametric shape deformation and image processing (DPSO method)	- use of ACSAmax and muscle length (ML) obtained using full muscle reconstruction and shape factor (p), volume: p* ACSAmax* ML —reduced MRI set method: model using the DPSO method, with 5 segmented slices, volume predicted from a multilinear regression	-	-	-	volume	concurrent validity	volume RMSE
** **	* *								
**Andrews 2015 [65]**	Gra, Sar, BFL, RF, ST, BFS, SM, VI, VM, Add, VL, left side	slice by slice manual segmentation	interactive segmentation using shape priors + statistical shape model	image preprocessing (linear transformation)	1, physical therapist, expert	-	3D shape	concurrent validity	DSI, mean Surf D
** **									
**Barnouin 2014[46]**	RF, VI, VL, VM, Qua, both sides	-	slice by slice manual segmentation, volume: muscle tissue area * interslice distance	-	2, trained	-	volume	inter rater reliability (muscle volume estimation, muscle individual contribution)	ICC, Student, mean diff
** **									
**Barnouin 2015 [47]**	RF, VI, VL, VM, both sides	slice by slice manual segmentation, volume: cylinder method	- slice by slice manual segmentation, volume: cone method/ 3d-order polynomial regression/ 4th-order polynomial regression '- manual segmentation of a reduced number of slices, volume: cylinder/ cone method/ 3d-order polynomial regression/ 4th-order polynomial regression	-	-	-	volume	concurrent validity, comparison between methods	ANOVA, mean diff, CV
** **									
**Belavy 2011 [55]**	RF, VM, VL, VI, Sar, Gra,Add M, Add L, BFL, BFS,ST, SM, GL, GM, So+FHL, TP, FDL, Per LBT, TA +EDL + EHL, left side	slice by slice manual segmentation, volume: linear interpolation	manual segmentation of a reduced number of slices, selection of the segmented slices with 5 algorithms including subalgorithms with various number of slices (1-largest CSA and the sum of the 3,6,9 … largest CSA measurement/ 2-largest CSA with immediately adjacent CSAs/ 3-same as 2 except every second images taken/ 4- method using CSA at 30, 40, 50, 80%/ 5- most proximal CSA with every 2d, 3d, 4th … CSA measurements), volume: linear interpolation	number of slices chosen: % within 0.5% of the reference % change in muscle volume; variability of the % change in muscle size same or less than that of the variability of the reference % change in muscle volume.	1, NR	-	volume change	concurrent validity	Pearson correlation coefficient r, mean percentage change
** **									
**Elliot 1997 [66]**	GM, GL, So	-	image based segmentation + manual segmentation, volume: addition of the number of voxels	correction algorithm for partial volume effect	2, trained	-	volume	inter rater reliability	correlation coefficient, max diff
** **	** **	** **	** **	** **	** **	** **	** **	** **	** **
**Eng 2007 [54]**	PT (10times), ECRB (10 times), EPL (10 times), FCU (7 times), BR (6 times)	dissection	manual segmentation in the 3 planes, volume: addition of the number of voxels	-	2, NR	-	volume	concurrent validity, inter rater reliability	ICC, mean diff
** **	* *								
**Engstrom 2011 [67]**	QL, Ps, ESM, both sides	slice by slice manual segmentation	atlas based + statistical shape based segmentation	image preprocessing (bias field correction, partial volume interpolation)	1, expert (for manual segmentation)		'3D shape	concurrent validity	DSI, TC, mean Surf D
** **	* *								
**Jolivet 2014 [68]**	RF, VLMI, Sar, TFL, BFS, BFL, ST, ST, Gra	slice by slice manual segmentation, volume: using 3D shape	segmentation using parametric shape deformation and image processing, improvements (improved DPSO technique: semi automatic contouring, automatic adjustements of the intermediate contours)	number of slices chosen to obtain an error <5%/ <5mm	-	-	volume, 3D shape	concurrent validity	point-to-surface distance 2*RMSE
** **	* *								
**Kim 2017 [29]**	Sspi	thresholding and manual post- processing	image based and shape based segmentation, volume: accumulation of the 2D contours, Laplacian smoothing process	-	2, experts	-	3D shape	concurrent validity	DSI, Accuracy = (RP+ RN)/ (RP+ E N+ E P+ RN), mean Surf D, Max Surf D
** **									
**Lehtinen 2003 [56]**	Sspi,Ssca, Ispi+Tmin	slice by slice manual segmentation, volume: calculated by the software	- single slice manual segmentation (at the Y-shaped position), volume: calculated by the software '- manual segmentation of 2 slices (at the Y-shaped position and at a defined more medial position), volume: calculated by the software	-	2, orthopaedic surgeons	each operator contoured muscles 3 times on 3 days	volume	concurrent validity, intra and inter rater reliability	Student, mean diff, 2SD, CV
** **	* *								
**Le Troter 2016 [48]**	RF, VI, VM, VL, Qua, right side	slice by slice manual segmentation, volume: cone method	- atlas based segmentation (semi automated) '- atlas based segmentation (fully automated)	-	1, experienced	-	volume, 3D shape	concurrent validity, repeatability, evaluation of affine and non linear registration methods, and fusion methods	ICC, CV, DSI, FNVF, FPVF, MVSF
** **				[53]					
**Lund 2002 [49]**	TA+EDL+EHL, left side	slice by slice manual segmentation, volume: cylinder method	- slice by slice manual segmentation, volume: NR —slice by slice manual segmentation, volume: cone method '- manual segmentation of 8 slices, volume: cylinder method '- manual segmentation of 8 slices, volume: cone method	number of slices chosen: to have < 10% difference/ reference volume	2, NR	manual segmentation of 13 slices equally distributed 3 times by one operator, 1 time by another	volume	concurrent validity (reduced number of slices), intra and inter rater reliability using 13 slices, comparison between methods	ICC, ANOVA, mean diff, 2SD
** **	* *								
**Marcon 2015 [9]**	Qua	manual segmentation of a reduced number of slices (every third slice), volume: NR	- single slice manual segmentation (at 25cm above the knee joint), volume: NR	slice at 25cm (rather than slice at 15 and 20cm) chosen: to have the minimal SEE	1, musculoskeletal radiology fellow	operator repeated the every third slice manual segmentation	volume	concurrent validity (single slice), intra rater reliability (every 3d slice manual segmentation)	ICC, SEE
** **									
**Mersmann 2014 [57]**	GM, GL, SO, TS, right side	slice by slice manual segmentation, volume: integral of the CSA along the muscle length	single slice manual segmentation (CSA max), muscle length (ML) obtained using full muscle reconstruction and shape factor (p) determined in a group with untrained and trained persons, volume: p* ACSAmax* ML	-	-	-	volume	concurrent validity	r^2^, ANOVA, volume RMSE
** **									
**Mersmann 2015 [58]**	VL, VM, VI, one side	slice by slice manual segmentation, volume: integral of the CSA along the muscle length	single slice manual segmentation (CSA max), muscle length (ML) obtained using full muscle reconstruction and shape factor (p), determined in a group with untrained and trained persons, volume: p* ACSAmax* ML	-	-	-	volume	concurrent validity	coefficient of determination r^2^, ANOVA, volume RMSE
** **									
**Moal 2014 [59]**	Add BLM, BF, ES, GlMa, GlMe, GlMi, Gra, Il, Obl, Ps, QL, RA, RF, Sar, SMT, TFL, VLI, VM	slice by slice manual segmentation (T1 images), volume using 3D shape	segmentation using parametric shape deformation and image processing (DPSO)	-	3 experienced operators	3 operators made 3 T1 reconstruction and 3 Fat reconstruction (using DPSO method)	'volume, 3D shape	- intra rater reliability and inter rater reliability of the DPSO method for T1 and fat images '- concurrent validity of the DPSO method for T1 and fat images with reference method	Student, mean diff, SD, CV, point to surface distance 2*RMSE
** **	* *								
**Morse 2007 [60]**	Qua, VL, VM, VI, RF, right side	slice by slice manual segmentation, volume: muscle tissue area * interslice distance	- 1/single slice manual segmentation (CSAmax), muscle length (ML) with US, volume: equation using ML and ACSA max '- 2/ single slice manual segmentation (CSA at 40% from the distal end of the femur), regression equation to estimate the maximum muscle cross-sectional area, muscle length (ML) with US, volume: equation using ML and ACSA max '- 3/same method as 2/ with CSA at 50% '- 4/same method as 2/ with CSA at 60%	-	1 (measures made 3 times, average recorded)	-	volume	concurrent validity	r^2^, SEE, mean diff, 1.96SD
** **									
**Nordez 2009 [27]**	Qua (VL+VI+VM+RF)	slice by slice manual segmentation, volume using 3D shape.	- manual segmentation of a reduced number of slices (3–21), volume: cone method, '- manual segmentation of a reduced number of slices (3–21), volume: Cavalieri formula '- manual segmentation of a reduced number of slices, cubic spline interpolation to estimate missing CSAs '- manual segmentation of a reduced number of slices (3–21), volume: DPSO	number of slices chosen to obtain an error< = 1.1%	2, NR	1st operator outlined all the slices a second time on different days.	volume	intra rater reliability and inter rater reliability for the reference technique, concurrent validity, comparison between methods	ICC, ANOVA, Student, mean diff, 1.96SD
** **									
**Popadic 2011 [50]**	TB, both sides	slice by slice manual segmentation, volume: cone method	- single slice manual segmentation (CSA max), humerus length (HL), volume: equation using CSAmax, humerus length (HL), BMI '- single slice manual segmentation (CSA max), humerus length (HL), volume: equation using CSAmax, HL '- single slice manual segmentation (CSA 50%), humerus length (HL), volume: equation using CSA50%, HL '- single slice manual segmentation (CSA 60%), humerus length (HL), volume: equation using CSA 60%, HL	-	-	-	volume, volume change	concurrent validity muscle volume, muscle volume change)	adjusted r^2^, RSE
** **									
**Skorupska 2016 [61]**	Pir, GlMi, GlMe, GlMa, both sides	-	slice by slice manual segmentation, volume: addition of the voxels and multiplication by the voxel dimension	-	2 physical therapists, 3/0 years of experience, trained	-	volume	inter rater reliability	ICC
** **									
**Smeulders 2010 [62]**	FCU, ECU, right side	-	slice by slice manual segmentation, volume: muscle tissue area * interslice distance	-	2, NR	1st operator repeated the evaluation of the first dataset, each observer evaluated both datasets	volume	intra rater reliability, inter rater reliability, repeatability	ICC, Student, mean diff, CV, SDD
** **	* *								
**Springer 2012 [48]**	GlMe, GlMi, OE, both sides	-	slice by slice manual segmentation, volume: NR	-	2, NR	2d operator repeated the evaluation of all datasets after an interval of 4 weeks.	volume	intra rater reliability, inter rater reliability	Student, mean diff, 1.96SD, CV
** **									
**Sudhoff 2009 [63]**	SM, ST, BFS, BFL, Sar, TFL, Gra, VLI, VM, RF, GM, GL	slice by slice manual segmentation (T1 images), volume: using 3D shape	segmentation using parametric shape deformation and image processing (DPSO)	number of slices chosen to obtain an error<5%	2, NR	-	volume, 3D shape	concurrent validity, inter rater reliability	ICC, mean diff, SD, point to surface distance error, point to surface distance 2*RMSE
** **	* *								
**Tingart 2003 [25]**	Sspi,Ssca, Ispi+Tmin	dissection, water displacement	slice by slice manual segmentation, volume: muscle tissue area * interslice distance	-	3, NR	each operator contoured muscles 3 times on 3 days	volume	concurrent validity, intra rater reliability, inter rater reliability	Pearson r^2^, mean diff, SD, CV
** **	* *								
**Tracy 2003 [26]**	Qua, trained side	slice by slice manual segmentation, volume: cone method	- manual segmentation of a reduced number of slices (every 2nd/ 4th/ 6ty/ 8th/ 10th section), volume: cone method '- single slice manual segmentation (CSAmax), volume: univariate regression	-	1, NR	-	volume, volume change	concurrent validity (muscle volume, muscle volume change after training)	r^2^, SEE, mean diff, 2SD
** **	* *								
**Valentin 2015 [45]**	ES, M, RA, Ps both sides	-	slice by slice manual segmentation, volume: muscle tissue area * interslice distance	-	2, novice (received training)	new analysis made 2 weeks later if low/ moderate agreement between assessors on the 1st evaluation	volume	inter rater reliability	ICC, mean diff, 2SD
** **	* *								
**Vanmechelen 2017 [51]**	GM, SOL, TA, RF, SM, ST, left side	slice by slice manual segmentation, volume: muscle tissue area * slice thickness	single slice manual segmentation, muscle length (ML) obtained using full muscle reconstruction and form factor (FF), volume: ((ACSAmax* ML)-Offset)*FF	-	-	-	volume	concurrent validity	r^2^, SEE
** **									
**Yamauchi 2017 [28]**	VL, VM, VI, RF, SM, ST, BFS, BFL, painful side	slice by slice manual segmentation, volume: muscle tissue area * interslice distance	-single slice manual segmentation at different femoral length levels, femoral length (FL), volume: regression equations which varied for each muscle '- use of muscle thickness at different femoral length levels and femoral length (FL), volume: regression equations which varied for each muscle	Use of the CSA at 60% from the distal end of the femur and muscle thickness at 50% of the distal end of the femur to have the best correlations with MV	1, trained image analyst	-	volume	concurrent validity	SEE

NR: not reported

ICC: intraclass correlation coefficient, mean diff: mean difference, SD: standard deviation, CV: coefficient of variation, SDD: smallest detectable difference, RMSE: root mean square error, SEE: standard error of the estimate, DSI: Dice similarity index, mean surf D: mean surface distance, max surf D: maximal surface distance, TC: Tannimoto coefficient, FNVF: false negative volume fraction, FPVF: false positive volume fraction, MVSF: muscle volume similarity fraction

RF: rectus femoris, VI: vastus intermedius, VL: vastus lateralis, VM: vatsus medialis, Qua: quadriceps, Pir: Piriformis, GlMi: Gluteus Minimus, GlMe: Gluteus Medius, GlMa: Gluteus Maximus, FCU: flexor carpi ulnaris, ECU: extensor carpi ulnaris, Sspi: Supraspinatus, Ssca: Subscapularis, Ispi+Tmin: Infraspinatus and Teres minor, ES: Erector Spinae, M: multifidus, RA: rectus abdominis, Ps: Psoas, Sar: Sartorius, Gra: Gracilis, AddM: Adductor Magnus, Add L: Adductor longus, BFL: Biceps Femoris Long head, BFS: Biceps Femoris Short head, ST: Semi Tendinosus, SM: Semi Membranosus, GL: Gastrocnemius Lateralis, GM: Gastrocnemius Medialis, So+FHL: Soleus and flexor hallucis longus, TP: Tibialis Posterior, FDL: flexor digitorum longus, Per LBT: Peroneus (Longus, Brevis, Tertius), TA+EDL+EHL: tibialis anterior and extensor digitorum longus and extensor hallucis longus, So: Soleus, TS: triceps surae, TB: triceps brachii, TA: Tibialis Anterior, VLMI: Vastus Lateralis and Medius and Intermedius, TFL: tensor Fascia Lata, Add BLM: adductor (brevis, longus, magnus), Il: Iliacus, Obl: Obliquus (transversus abdominis, internus and externus obliquus), QL: Quadratus Lumborum, VLI: Vastus Lateralis and Intermedius together, VLMI: Vastus Lateralis and Medialis and Intermedius, BF: Biceps Femoris, SMT: Semi Membranosus and Tendinosis, ESM: erector spinae and multifidus, PT: pronator teres, ECRB: Extensor Carpi Radialis Brevis, EPL: Extensor Pollicis Longus, Br: Brachioradialis

## Results

### Selection process

**Fig 1:**
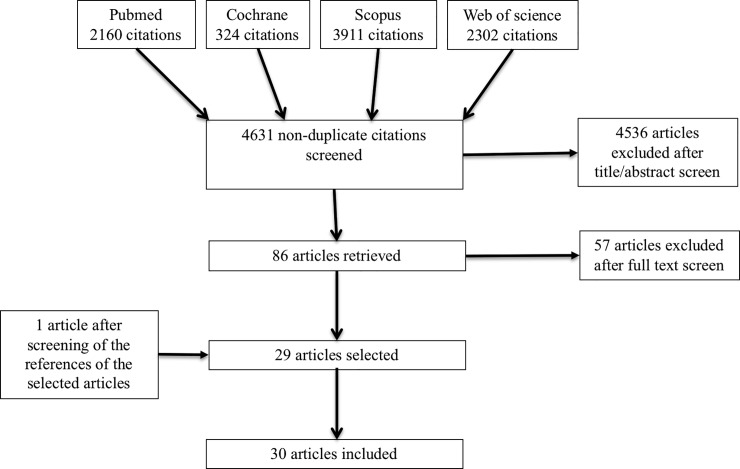
Flow chart.

The literature search identified 2160 citations in PubMed, 324 citations in the Cochrane Library, 3911 citations in Scopus, 2302 citations in Web of Science. After removing duplicates, 4631 remained. After screening titles and abstracts, 86 articles were found to be potentially eligible. Finally, 30 met the inclusion criteria and were included (Fig 1).

### Quality assessment

The mean Q score of the articles included was 64.1/100 (SD: 9.7). The primary aim of seventeen articles was to determine the metrological properties of a measurement technique. Ten articles had a score above 70/100 [9,27,28,45–51], fifteen articles had a score between 60 and 70/100 [25,26,52–64] and five articles had a score below 60/100 [29,65–68]. Details of the scores of each article are provided in S2 Table.

### Description of studies

The methodological characteristics (samples, designs and measurement methods) of each article are presented in Table 1 and S3 Table.

The articles included primarily focused on segmentation techniques. Manual techniques (including slice by slice cross sectional area (CSA) segmentation, segmentation of CSA in a reduced number of slice(s), segmentation of CSA on one slice and muscle length use) were evaluated in twenty-four articles [9,25–28,45–58,60–64] and partially or completely automatic segmentation techniques (deformation of a parametric specific object, semi-automated and automated atlas-based, image-based and shape-based, atlas-based and statistical shape-based, and interactive-segmentation using shape priors and statistical shape modelling methods) were evaluated in ten articles [27,29,48,53,59,64–68].

Muscle volume was evaluated in twenty-six articles [9,25–28,45–54,56–64,66,68], changes in muscle volume were evaluated in three articles [26,50,55] and 3D shapes were evaluated in seven articles [29,48,59,64,65,67,68].

Seventeen articles included only healthy subjects [26,27,45–50,52,53,57–60,62,64,67] and six included subjects with a muscular pathology of which five were on adults with: low back pain, total unilateral arthroplasty, anterior cruciate ligament reconstruction, chronic obstructive pulmonary disease or knee osteoarthritis [9,28,61,63,65]; the fifth article was on children with cerebral palsy [51]. Three articles evaluated cadavers [25,54,56].

Forty different muscles were examined: upper limb muscles were assessed in six articles [25,29,50,54,56,62] and lower limb muscles in twenty-four articles [9,26–28,45–49,51–53,55,57–61,63–68]. The rectus femoris muscle was the most frequently evaluated (n = 13 articles). Different functional groups were used (n = 18 articles). For example, for the quadriceps, it could be considered as a whole, or groupings could be made between vastus lateralis and intermedius or vastus lateralis, medialis and intermedius, or all the muscles could be segmented separately.

With regards to MRI parameters, 1.5T scanners were the most frequently used (n = 22 articles), T1 weighted sequences were used in twenty four articles and 3D sequences were used in seven articles [9,29,46,47,52,54,66]. Axial slices were the most frequently segmented.

### Manual techniques (Tables 1 and 2, S3, S4 and S5 Tables)

**Table 2:** Evidence of validity and reliability by technique and by muscle.

		slice-by-slice CSA segmentation	CSA segmentation on a reduced number of slices	CSA segmentation/ muscle thickness using a single slice and muscle length	CSA segmentation on a single slice	deformation of a parametric specific object (DPSO)	deformation of a parametric specific object (DPSO), reduced MRI set method	other automatic methods
**supraspinatus**	validity	++ [25]	+ (2 MSS) [56]		+ [56]			++++ [29]
** **	intraR	+++ [25]	+++ (2 MSS) [56]		+++ [56]			
** **	interR	+++ [25]	+++ (2 MSS) [56]		+++ [56]			
**subscapularis**	validity	++ [25]	++ (2 MSS) [56]		+ [56]			
	intraR	+++ [25]	+++ (2 MSS) [56]		+++ [56]			
	interR	+++ [25]	+++ (2 MSS) [56]		+++ [56]			
**infraspinatus + teres minor**	validity	++ [25]	++ (2 MSS) [56]		++ [56]			
	intraR	+++ [25]	+++ (2 MSS) [56]		+++ [56]			
** **	interR	+++ [25]	++ (2 MSS) [56]		+++ [56]			
**triceps brachii**	validity			++ [50]				
**flexor carpi ulnaris**	intraR	++++ [62]						
	interR	+++ [62]						
**extensor carpi ulnaris**	intraR	+++ [62]						
	interR	++ [62]						
**quadratus lumborum**	validity			+ [53]		++ (Dixon)/ +++ (T1) (18%MSS) [59]		+++ [67]
	intraR					+++ (18%MSS) [59]		
	interR					+++ (18%MSS) [59]		
**erector spinae**	validity			++ [53]		++ (Dixon)/ +++ (T1) (15% MSS) [59]		
	intraR					+++ (15% MSS) [59]		
	interR	+++ [45]				+++ (15% MSS) [59]		
**multifidus**	interR	++ [45]						
**rectus abdominis**	validity					++ (Dixon)/ +++ (12% MSS) (T1) [59]		
	intraR					+++ (12% MSS) [59]		
	interR	+ [45]				++ (12% MSS) [59]		
**psoas**	validity					++ (Dixon)/ +++ (T1) (10% MSS) [59]		+++ [67]
	intraR					+++ (10% MSS) [59]		
	interR	+++ [45]				+++ (10% MSS) [59]		
**erector spinae and multifidus**	validity							+++ [67]
	interR	+++ [45]						
**gluteus medius**	validity			++ [53]		++ (Dixon)/ +++ (T1) (25% MSS) [59]		
	intraR	+++ [63]				+++ (25% MSS) [59]		
	interR	+++ [61] + [63]				++ (25% MSS) [59]		
**gluteus minimus**	validity			+ [53]		++ (Dixon)/ +++ (T1) (30% MSS) [59]		
	intraR	+ (P) / ++ (H) [63]				++ (30% MSS) [59]		
	interR	+++ [61] + [63]				++ (30% MSS) [59]		
**external obturator**	intraR	++ [63]						
	interR	+ [63]						
**gluteus maximus**	validity			++ [53]		++ (Dixon)/ +++ (T1) (18% MSS) [59]		
	intraR					+++ (18% MSS) [59]		
	interR	+++ [61]				+++ (18% MSS) [59]		
**piriformis**	interR	+++ [61]						
**iliacus**	validity					++ (Dixon)/ +++ (T1) (25% MSS) [59]		
	intra reliab					+++ (25% MSS) [59]		
	inter reliab					+++ (25% MSS) [59]		
**obliquus**	validity					++ (Dixon)/ +++ (T1) (20% MSS) [59]		
	intraR					+++ (20% MSS) [59]		
	interR					+++ (20% MSS) [59]		
**spine flexors**	validity						++ [53]	
**spine extensors**	validity						+ [53]	
**hip flexors**	validity						++ [53]	
**hip extensors**	validity						++ [53]	
**rectus femoris**	validity		++++ (alg 2, 9 MSS) [55] + to +++ (depending of nr of MSS) [47]	+++ [51] ++ [60] + (CSA/thickness) [28] +++ [53]		++ (Dixon)/ +++ (T1) (13% MSS) [59] ++ (6 MSS) [64] ++ (improved DPSO, 5 MSS) [68]		+ (fully)/ +++ (semi) [48] +++ [65]
	intraR					+++ (13% MSS) [59] ++ 6 MSS)[64]		
	interR	++ (1 subject) [64] ++ [46] +++ [48]				+++ (13% MSS) [59]		
**vastus lateralis**	validity		+ to +++ (depending of nr of MSS) [47]	+++ (58) +++ [60] + (thickness)/ ++ (CSA) [60]				+ (fully)/ +++ (semi) [48] +++ [65]
	interR	++ [48] ++ [46]						
**vastus medialis**	validity		+ to +++ (depending of nr of MSS) [47]	++ [53] +++ [60] ++ (58) + (thickness)/ ++ (CSA) [60]		++ (Dixon)/ +++ (T1) (15% MSS) [59] ++ (7 MSS) [64]		+++ (fully and semi) [48] +++ [65]
	intraR					+++ (15% MSS) [59] ++ (7 MSS) [64]		
	interR	++ [46] ++ (1 subject) [64] +++ [48]				+++ (15% MSS) [59]		
**vastus intermedius**	validity	++++ Barnouin 2015	+ to +++ (depending of nr of MSS) [47]	++ (58) ++ [60] + (thickness)/ ++ (CSA) [60]				+ (fully)/ +++ (semi) [48] +++ [65]
	interR	+++ [46] +++ [48]						
**vastus lateralis and intermedius**	validity			+++ [53]		++ (Dixon)/ +++ (T1) (15% MSS) [59] ++ (7 MSS) [63]		
	intraR					+++ (15% MSS) [59] +++ (7 MSS) [63]		
	interR	++ (1 subject) [64]				+++ (15% MSS) [59]		
**vastus lateralis and medialis and intermedius**	validity		++++ (alg 3, 3 MSS) [55]			++ (improved DPSO, 5 MSS) [68]		
**quadriceps**	validity		++ to ++++ depending of nt of MSS) [26] + to +++ (depending of nt of MSS) [47] +++ to ++++ (depending of volume calculation method, 5 to 12 MSS) [27]	+ to ++ (CSA at different levels) [60]	++ [26] ++ (9)	++++ [27]	++ [53]	
	intraR	++++ [27]						
	interR	+++ [48] ++ [46] ++++ [27]	+++ (9)					
**sartorius**	validity		++++ (alg 3, 7 MSS) [55]	+++ [53]		++ (Dixon)/ +++ (T1) (10% MSS) [59] ++ (7 MSS) [64] ++ (improved DPSO, 5 MSS) [68]		+++ [65]
	intraR					+++ (10% MSS) [59] + (7 MSS) [64]		
	interR	+ (1 subject) [64]				+++ (10% MSS) [59]		
**tensor fascia lata**	validity			++ [53]		++ (Dixon)/ +++ (T1) (15%MSS) [59] ++ (6 MSS) [64] ++ (improved DPSO, 4 MSS) [68]		
	intraR					+++ (15%MSS) [59] ++ (6 MSS) [64]		
	interR	++ (1 subject) [64]				+++ (15%MSS) [59]		
**biceps femoris short head**	validity		++++ (all) [55]	++ [53] + [60]		++ (8 MSS) [64] ++ (improved DPSO, 5 MSS) [68]		+++ [65]
	intraR					+ (8 MSS) [63]		
	interR	+ (1 subject) [64]						
**biceps femoris long head**	validity		++++ (all) [55]	++ [53] + [60]		+++ (6 MSS) [64] ++ (improved DPSO, 4 MSS) [68]		+++ [65]
	intraR					+ (6 MSS) [64]		
	interR	++ (1 subject) [64]						
**biceps femoris**	validity					++ (Dixon)/ +++ (T1) (12% MSS) [59]		
	intraR					+++ (12% MSS) [59]		
	interR					+++ (12% MSS) [59]		
**semi tendinonsis**	validity		++++ (alg 2, 11 MSS) [55]	++ [53] + [60] ++ [51]		++ (6 MSS) [64] ++ (improved DPSO, 6 MSS) [68]		+++ [65]
	intraR					+ (6 MSS) [64]		
	interR	++ (1 subject) [64]						
**semi membranosus**	validity		++++ (all) [55]	++ [53] + [60] ++ [51]		++ (6 MSS) [64] ++ (improved DPSO, 5 MSS) [68]		+++ [65]
	intraR					+ (6 MSS) [64]		
	interR	++ (1 subject) [64]						
**gracilis**	validity		++++ (all) [55]	++ [53]		++ (Dixon)/ +++ (T1) (10% MSS) [59] ++ (7 MSS) [64] ++ (improved DPSO, 4 MSS) [68]		+++ [65]
	intraR					+++(10% MSS) [59] + (7 MSS) [64]		
	interR	+ (1 subject) [64]				+++ (10% MSS) [59]		
**AddOP**	validity			++ [53]				
**add longus**	validity		++++ (alg 2, 3 MSS) [55]					
**add magnus**	validity		++++ (alg 2, 1 MSS) [55]					
**semi membranosus and tendinosis**	validity					++ (Dixon)/ +++ (T1) (11%MSS) [59]		
	intraR					+++ (11%MSS) [59]		
	interR					+++ (11%MSS) [59]		
**adductor (brevis, longus, magnuas)**	validity					++ (Dixon)/ +++ (T1) (20%MSS) [59]		
	intraR					+++ (20%MSS) [59]		
	interR					+++ (20%MSS) [59]		
**adductor**	validity							+++ [65]
**knee flexors**	validity						++ [53]	
**knee extensors**	validity						++ [53]	
**gastrocnemius medialis**	validity		++++ (alg 2, 2 MSS) [55]	++ [52] ++ [51] +++ [57]		++ (8 MSS) [64]		
	interR	++ (1 subject) [64]						
**gastrocnemius lateralis**	validity		++++ (alg 2, 7 MSS) [55]	+ [52]		++ (6 MSS) [64]		
	intraR					+ (6 MSS) [64]		
	interR	++ (1 subject) [64]						
**soleus**	validity		++++ (alg 2, 4 MSS) [55]	++ [52] ++ [51] ++ [57]				
**tibialis posterior**	validity		++++ (alg 2, 12 MSS) [55]					
**peroneus (Longus, Brevis, Tertius)**	validity		++++ (alg 3, 7 MSS) [55]					
**tibialis anterior + extensor digitorum longus + extensor hallucis longus**	validity		++++ (alg 3, 10 MSS) [55] ++++ (8 MSS) [49]					
	intraR		+++ 13 slices [49]					
	interR		++ 13 slices [49]					
**flexor digitorum longus**	validity		++++ (alg 2, 7 MSS) [55]					
**tibialis anterior**	validity			++ [51]				

IntraR: intra rater reliability, interR: inter rater reliability

Excellent, good, moderate and poor metrological qualities are represented by ++++, +++, ++ and + signs respectively

#### Slice by slice CSA segmentation

Estimation of muscle volume using slice-by-slice CSA segmentation was evaluated in 11 articles (Range Q score: 61–73, mean Q score: 68.5 [25,27,45–49,61–64]). Slice thicknesses varied between 1.5mm [25] and 10 mm [45]. In seven of those articles, there were no gaps between slices [25,27,46,47,49,61,64]. After segmentation, seven different calculation methods were used to estimate muscle volume.

Moderate to good validity was found between manual slice-by-slice CSA segmentation and measurements from cadavers (n = 1 article, [25]). Intra-rater reliability was good to excellent (n = 4 [25,27,62,63]). Inter-rater reliability was moderate to good (n = 8 [25,27,44,45,60–63]). Test retest reliability was good (n = 2, [48,62]). Results were less reliable for external obturator volume [63] or gluteus minimus volume. Results for quadriceps volume were more reliable than results for the individual muscles that constitute it [27,46,48,64]. In articles that included both healthy and pathological muscles, results were more reliable for healthy muscles than pathological muscles [61,63]. Mean differences of less than 1% were found between different methods of volume estimation (cone, cylinder, 3^rd^ and 4^th^ order polynomial regression equations) (n = 2, [47,49]).

#### CSA segmentation on a reduced number of slices

Estimation of muscle volume using CSA segmentation on a reduced number of slices was evaluated in 6 articles (Range Q score: 66–73; mean Q score: 70.2 [9,26,27,47,49,55]). The choice of slices for segmentation was based on different elements, such as the number of slices [27,49], interslice distance [9,26,47,55], specific characteristics of the slices (for example slices with largest CSA, or slices taken in a specific part of the muscle [55]). Six different methods of volume estimation were reported: the cylinder method [49], the cone method [26,27,49], the Cavalieri method [27], cubic spline interpolation [27], and 3^rd^ and 4^th^ order polynomial equations [47]. Comparison between segmentation data from techniques using a reduced number of slices and slice-by-slice segmentation (n = 5 [26,27,47,49,55]) showed that validity varied from poor to excellent. Validity was excellent when a sufficient number of slices was segmented Reducing the number of slices systematically increased the error. The number and the choice of slices to segment and the choice of volume calculation method to obtain a pre-determined error was specific to each muscle. The method of CSA segmentation on a reduced number of slices had moderate to good intra and inter-rater reliability ICC (n = 2 [9,49]).

#### CSA segmentation or muscle thickness using a single slice and muscle length

Eight articles evaluated the use of CSA segmentation or muscle thickness using a single slice and muscle length to estimate muscle volume (Range Q scores: 61–78; mean Q score: 67.4) [50–53,57,58,60]. For the measurements, either the slice with the greatest CSA [51–53,57,58,60], or slices taken at specific locations (for example at 50% of the bone length) [28,50,60] were used. To estimate muscle volumes, equations using muscle length, CSA and shape factors were used. The validity of these methods was evaluated by comparing with slice-by-slice manual segmentation in all the studies but one. Results showed that validity ranged from poor to good, but was mostly moderate (n = 8 [50–53,57,58,60]). The smallest errors were found for CSA measured at 60% from the distal end of the femur or humerus for the quadriceps, knee flexors and triceps brachialis (n = 3 [28,50,60]), and using muscle thickness at 50% of the femur for the quadriceps (n = 1 [28]). Some muscle volumes appeared to be more difficult to obtain with CSA segmentation using a single slice, such as gluteus minimus and quadratus lumborum, for which validity was poor. No studies evaluated reliability.

#### CSA segmentation on a single slice without muscle length

Estimation of muscle volume using CSA segmentation on a single slice without muscle length was evaluated in three articles (Range Q score: 68–72; mean Q score: 69.3 [9,26,56]). Specific slices were chosen, either the one with the largest CSA [26] or those taken at specific locations [9,56]. Manual slice-by-slice segmentation was used as the control reference to evaluate validity, and showed that it was poor to moderate (n = 3 [9,26,56]). Poor results were found for supraspinatus, and subscapularis muscles [56]. Intra and inter-rater reliability were good (n = 1 [56]).

### Automatic segmentation techniques (Tables 1 and 2, S3, S4 and S5 Tables)

#### Deformation of a parametric specific object method with manual segmentation

Estimation of muscle volume and/or 3D shape using the deformation of a parametric specific object (DPSO) method with manual segmentation was evaluated in five articles (Range Q score: 46–71; mean Q score: 62.2 [27,53,59,64,68]).This technique involves manual contouring on a reduced set of images, followed by a parametric shape-based interpolation combined with a kriging technique in order to obtain a surface model without using the intermediate slices [68,69]. Validity was moderate to good compared to slice-by-slice manual segmentation (n = 4 [27,59,64,68]). Reducing the number of slices increased the error (n = 1 [53]). Reliability was poor to good depending on the muscle (n = 2, [59,64]). The number of manually segmented slices required to obtain a pre-determined error was specific to each muscle. A larger number of slices was necessary for gluteus minimus, gluteus medius, obliquus and iliacus.

#### Other automatic segmentation techniques

Four other methods to estimate 3D muscle shapes were evaluated: semi-automated and automated atlas-based segmentation [48], image-based and shape-based segmentation [29], atlas-based and statistical shape-based segmentation [67], and interactive-segmentation using shape priors and statistical shape modelling [65] (Range Q scores: 49–73: mean Q score: 55.5). Andrews et al. used a probabilistic shape representation called generalized log-ratio representation that included adjacency information along with a rotationally invariant random forest boundary detector to automatically segment thigh muscles [65]. Kim et al. used an active contour segmentation method with a level sets approach to automatically extract supraspinatus muscle from an MR image [29]. Engstrom et al., used a statistical shape model (SSM) to automatically segment quadratus lumborum [67]. During the fitting process, the deformable SSM was constrained using probabilistic MR atlases. Le Trotter et al. used a multi-atlas based automatic segmentation method to quantify the volume of the quadriceps femoris muscle group [48]. Validity against slice by slice manual segmentation was moderate to excellent and most of the results showed good validity. No studies of reliability were found.

### Technique feasibility (S4 Table)

The duration of segmentation was evaluated in eight studies [25,26,46,56,59,64–66]. Use of a reduced number of slices to obtain muscle volume divided segmentation time by 4, use of only one or two slices divided segmentation time by 26 and 15, respectively [56]. Use of the DPSO method to evaluate 3D shape halved the time taken in one article [59] and divided it by 12 in another [27]. Using automatic segmentation methods, one article reported that the time-to-run, without human interaction, was about 50 minutes per image [65]. No other studies evaluated feasibility.

## Discussion

This review included 30 articles which primarily focused on segmentation techniques. It has reported currently available evidence for the metrological qualities of manual and automatic segmentation techniques that estimate muscle volume and shape, and the feasibility of their use in a clinical or research setting. The majority of studies reviewed included healthy subjects, evaluated lower limb muscles and used slice-by-slice manual segmentation as the gold-standard reference. Greater errors in volume and shape estimation were found to be produced by methods that simplified and shortened the manual segmentation process. Sufficient evidence was available to support the validity of the DPSO technique. A lack of robust studies meant that other automatic segmentation techniques could not be validated but the evidence currently available was considered to be encouraging and further work on these methods is indicated. Some particularities for specific muscles and segmentation techniques were highlighted.

### Metrological qualities of manual and automatic techniques

#### Manual segmentation techniques

Slice-by-slice manual segmentation was the most evaluated technique but its validity was only evaluated in one study (on rotator cuff muscles). As slice-by-slice manual segmentation is widely used as a reference method, further studies are warranted to confirm its validity. With regards to reliability, results varied among muscles. The use of different volume calculation methods did not seem to change the errors, indicating that errors found between measurements were likely related to segmentation. The quality of the results was lower for deep muscles such as gluteus minimus and for muscles whose boundaries are unclear, such as the individual muscles of the quadriceps. Identifying their external borders appears challenging. To limit these segmentation errors, we believe that it is essential that standardized procedures using clear anatomical landmarks per muscle are developed and implemented [46]. Despite the fact that few studies evaluated image acquisition methods, they appear to be key for the limitation of segmentation errors [70]. Regarding the studies that compared data from subjects with healthy or pathological muscles, the weaker reliability for pathological muscles could be attributed to shape changes and boundaries that are more difficult to identify [65]. Slice-by-slice manual segmentation is also time-consuming, hence it cannot be easily used in clinical practice.

Techniques based on the manual segmentation of a reduced number of slices reached good to excellent validity when a sufficient number of slices was segmented. The appropriate number of slices varied among muscles. For most, fewer than half of the total number of slices need to be manually segmented, with slice thicknesses of 10 mm and interslice distances of 5 mm, allowing shorter processing time, whilst maintaining an almost equivalent level of performance compared to slice by slice segmentation. Results can further be improved by the choice of appropriate slices to segment [55]. Errors in volume estimation can however occur when the number of segmented slices is reduced [26,27,47,49,55]. We were unable to determine any general rules based on muscle shape or the size, thus further studies are required to assess these methods in muscles that were not evaluated in this systematic review, especially upper limb and trunk muscles. Lastly, important differences between volume calculation methods were also highlighted. For example, the cone method was inappropriate for fusiform muscles [27,47].

Use of even faster techniques, such as the segmentation of a single slice with or without muscle length, could be associated with a loss of precision. Because of their speed of realization, these techniques can be used in clinical practice if the aim is, for example, to estimate the degree of muscle loss in diseases that causes severe atrophy, where differences of more than 10% in volume would normally be expected. Special attention must however be paid when using these methods for non-fusiform muscles. Although the guidelines used for the choice of each slice were detailed for each technique, there was little reliability evaluations. It has been previously reported that the optimal location of measurements can be difficult to both define and reproduce [61] thus there is a potential for errors to occur from manual CSA segmentation. Further studies are warranted to evaluate reliability.

#### Automatic segmentation techniques

The DPSO method, which involves automatic segmentation of intermediate slices, had good validity if enough slices were manually segmented. For non-fusiform and small muscles, a greater number of slices has to be manually segmented to maintain good accuracy. If this method is found to be reliable, it could be used in association with manual techniques to reduce the number of manually segmented slices and help save time. Further studies are warranted to determine which technique is the most accurate and fast between manual segmentation of a reduced number of slices with different volume estimation methods and manual segmentation with DPSO [27]. The results could differ depending on the muscles, because of their specific shapes and localizations.

The validity of the other four partially or completely automatic techniques analysed (semi-automated and automated atlas-based segmentation, image based and shape-based segmentation, atlas based and statistical shape-based segmentation) could not be confirmed in this review due to the small number of low-quality studies currently available, however it is important to note that results were encouraging. These techniques appeared to be promising in terms of validity. High quality, additional metrological studies are thus needed to validate them. Each technique had its own characteristics: segmentation using generalized log-ratio representation transformation can impose soft constraints whereas deformable statistical shape models and atlas-based segmentations use hard constraints. However, the generalized log-ratio representation method cannot effectively delineate pose variability as against the other techniques and thus requires image pre-processing as an additional step. Thus, some techniques may be more appropriate than others depending on the muscles and their properties and on the characteristics of the population (children, persons with muscle pathology etc.). Other findings indicated that techniques, such as random-walk segmentation [71,72], wavelet-based segmentation [73], or deep learning-based segmentation [74] should additionally be investigated further to determine if they could provide rapid, accurate, valid and reliable measurements of muscle volume and shape for use in routine clinical practice.

### Pathological muscles

Methods to estimate skeletal muscle volumes and/or 3D muscle shapes using MRI data are used clinically for diagnosis [14], to evaluate the effects of treatment [12], and as an aid to preoperative planning [18]. In the case of muscle pathologies, changes in muscle shape and signal occur because of muscle degeneration, which can render identification of muscle boundaries in MRI difficult (due to fatty and fibrous infiltration) [16,17]. Modification of the anatomical landmarks used for CSA segmentation, of techniques that are based on shape factors, and of volume estimation methods may therefore be required. This is, however, currently unknown due to a lack of studies that have evaluated pathological muscles. This finding suggested that specific metrological studies are required depending on the pathology being investigated in order to avoid measurement errors and that caution must be applied when extrapolating the results of techniques used in healthy muscles to those with pathologies.

### Image acquisition

The MRI protocol used to acquire images can have a huge impact on segmentation outcomes [70]. The studies included in this review mostly used T1 weighted sequences, suggesting that these anatomical sequences are appropriate for segmentation because of their ability to provide good quality images of the muscles, to distinguish the margins between them and because of their capacity to contrast bones from muscle [9,27,29,64]. However other sequences could also be used and differences in metrological properties between sequences were shown in one article [59]. No other studies compared different sequences in the articles included. Thus, data regarding the validity of the different sequences are warranted [59]. Regarding the issue of 2D or 3D acquisition, of the seven articles which used 3D sequences, none showed that 3D sequences yielded better results than 2D sequences. Most of them evaluated manual segmentation techniques. Since 3D sequences take longer to acquire, have lower contrast and are more sensitive to susceptibility and B0 inhomogeneities [75], there was no evidence to recommend 3D acquisition for manual segmentation. Continuous slice acquisition, allowing muscle tracking, might be an interesting method [55]. The size of the muscle should be considered in determining the resolution to use to avoid partial volume artefacts [49,66]. A greater resolution is needed for small muscles. We suggest the use of a T1 sequence, 2D acquisition with continuous slices between 1 and 10mm thick, oriented in an orthogonal way to the large axis of the muscles, with a resolution that avoids partial volume effects. However, the paucity of data in the articles included in the systematic review does not allow strong recommendations to be made. Lastly, no data are currently available to show the effect of MRI scanner and coil type on data acquisition and the quality of metrological parameters, despite the fact that all of these elements could impact on the accuracy and reliability of the muscle volume and shape [54,65,76,77]. Further studies are therefore warranted to clarify these issues.

The feasibility of MRI can be limited by the availability of MRI scanners and the cost of MRI devices and assessments. Thus, some other techniques, for example using ultrasonography, could be interesting to estimate skeletal muscle volumes and 3D muscle shapes [78].

### Improving future metrological study methodology

We believe future work should include evaluation of test-retest reliability since we found only two articles that assessed this [48,62]. Test retest reliability refers to the extent to which the rating of one sample of individuals by one observer on two or more separate occasions using the same test yields similar results, with all test conditions remaining as constant as possible [31]. This is of high importance because factors such as patient positioning could impact on the accuracy and reliability of the muscle volume and shape as determined using MRI [54,65,76,77]. The second evaluation of great importance in future work is responsiveness. Responsiveness refers to the quality of a measure when showing changes [32], and is also a very important quality for the evaluation of neuromuscular disease progression [10,11] and the effects of treatment [12,50]. We were unable to report on the responsiveness of techniques in the present review as it was only evaluated in two articles.

Furthermore, precise reporting of the statistical analysis method employed is essential for metrological studies. As a result of the work undertaken in this review we recommend that the following evaluations are included as standard in future work, in addition to the usual analyses of correlation to improve the internal validity, on measurement technique studies [30,79]. The first evaluation we recommend is measurement error. In order to demonstrate the reliability of a technique, the standardized error of measurement, including limits of agreement or smallest detectable change [30], should be known as they indicate whether the observed difference is due to a true change in muscle volume or size, or if it is simply a measurement error.

### Limitations

When considering our findings and recommendations, it is important to note that the strength of any conclusions depends on the quality of the original articles [43]. The articles were rated as moderate to good quality, however only two included statistical power calculations, reducing the conclusions that can be drawn from the results. This aspect of study design should be included in all future studies into this topic. A second limitation of this study is the heterogeneity of source material included, in particular the different MRI parameters used in the studies and the different muscles evaluated prevented pooled analysis from being carried out and complicated the synthesis of the results. Regarding MRI, even when the same sequences were used, the parameters remained heterogeneous since they were device-dependent. Regarding muscles, some muscles have been the focus of many studies, whilst others have been neglected. Clinicians and researchers should bear this in mind when using a technique that has not been previously evaluated for the muscle in question. The results of this study are therefore only relevant for the methods of estimation of muscle volume and shape evaluated by the studies included, and must be generalised with caution to other methods and other muscles. Finally, the statistical methods employed by the different studies also varied considerably which, in turn, further prevented more definite conclusions from being drawn in this review. The different statistical methods used to report concurrent validity (including r^2^, ICC, Dice Similarity Index), Tannimoto coefficient, mean differences, SD, SEE, RMSE and point-to-surface distance) and reliability (such as ICC, mean differences, RMSE, coefficient of variation and standard deviation) limited the synthesis of the data with a quantitative pooled analysis. Future work should aim to overcome as far as possible such diversity in order to both strengthen results as well as improving the generalisability of findings across different methods.

### Conclusion

The results of this systematic review provide a rationale for the choice of appropriate segmentation techniques depending on the muscle, the need for precision and the available time. Such uses could include diagnosis of a disease, evaluation of a treatment response, monitoring of disease progression or measurement for research purposes. Further research is required to confirm the validity of manual slice-by-slice segmentation and automatic techniques, except for DPSO for which there is sufficiently strong supporting evidence. The reliability of most techniques in current use also needs to be confirmed, except for manual slice-by-slice segmentation, which has been shown to be sufficiently reliable (if time consuming). Studies to evaluate different MRI protocols are warranted. Specific studies in pathological muscles are also needed to enable the proper application of such techniques in routine clinical practice.

## Supporting information

S1 Table Prisma checklist. (DOC)

S2 Table Quality assessment of the articles included in the review. (DOCX)

S3 Table Description of the population and MRI technique in the articles included. F: female, M: male, SD: standard deviation, NR: not reported
RF: rectus femoris, VI: vastus intermedius, VL: vastus lateralis, VM: vatsus medialis, Qua: quadriceps, Pir: Piriformis, GlMi: Gluteus Minimus, GlMe: Gluteus Medius, GlMa: Gluteus Maximus, FCU: flexor carpi ulnaris, ECU: extensor carpi ulnaris, Sspi: Supraspinatus, Ssca: Subscapularis, Ispi+Tmin: Infraspinatus and Teres minor, ES: Erector Spinae, M: multifidus, RA: rectus abdominis, Ps: Psoas, Sar: Sartorius, Gra: Gracilis, AddM: Adductor Magnus, Add L: Adductor longus, BFL: Biceps Femoris Long head, BFS: Biceps Femoris Short head, ST: Semi Tendinosus, SM: Semi Membranosus, GL: Gastrocnemius Lateralis, GM: Gastrocnemius Medialis, So+FHL: Soleus and flexor hallucis longus, TP: Tibialis Posterior, FDL: flexor digitorum longus, Per LBT: Peroneus (Longus, Brevis, Tertius), TA+EDL+EHL: tibialis anterior and extensor digitorum longus and extensor hallucis longus, So: Soleus, TS: triceps surae, TB: triceps brachii, TA: Tibialis Anterior, VLMI: Vastus Lateralis and Medius and Intermedius, TFL: tensor Fascia Lata, Add BLM: adductor (brevis, longus, magnus), Il: Iliacus, Obl: Obliquus (transversus abdominis, internus and externus obliquus), QL: Quadratus Lumborum, VLI: Vastus Lateralis and Intermedius together, VLMI: Vastus Lateralis and Medialis and Intermedius, BF: Biceps Femoris, SMT: Semi Membranosus and Tendinosis, ESM: erector spinae and multifidus, PT: pronator teres, ECRB: Extensor Carpi Radialis Brevis, EPL: Extensor Pollicis Longus, Br: Brachioradialis
FOV: Field of View, NEX: Number of Excitations, TR: Repetition Time, TE: Time to echo.
(DOCX)

S4 Table Metrological properties of techniques. ICC: intraclass correlation coefficient, mean diff: mean difference, SD: standard deviation, CV: coefficient of variation, SDD: smallest detectable difference, RMSE: root mean square error, SEE: standard error of the estimate, DSI: Dice similarity index, mean surf D: mean surface distance, max surf D: maximal surface distance, TC: Tannimoto coefficient, FNVF: false negative volume fraction, FPVF: false positive volume fraction, MVSF: muscle volume similarity fraction
RF: rectus femoris, VI: vastus intermedius, VL: vastus lateralis, VM: vatsus medialis, Qua: quadriceps, Pir: Piriformis, GlMi: Gluteus Minimus, GlMe: Gluteus Medius, GlMa: Gluteus Maximus, FCU: flexor carpi ulnaris, ECU: extensor carpi ulnaris, Sspi: Supraspinatus, Ssca: Subscapularis, Ispi+Tmin: Infraspinatus and Teres minor, ES: Erector Spinae, M: multifidus, RA: rectus abdominis, Ps: Psoas, Sar: Sartorius, Gra: Gracilis, AddM: Adductor Magnus, Add L: Adductor longus, BFL: Biceps Femoris Long head, BFS: Biceps Femoris Short head, ST: Semi Tendinosus, SM: Semi Membranosus, GL: Gastrocnemius Lateralis, GM: Gastrocnemius Medialis, So+FHL: Soleus and flexor hallucis longus, TP: Tibialis Posterior, FDL: flexor digitorum longus, Per LBT: Peroneus (Longus, Brevis, Tertius), TA+EDL+EHL: tibialis anterior and extensor digitorum longus and extensor hallucis longus, So: Soleus, TS: triceps surae, TB: triceps brachii, TA: Tibialis Anterior, VLMI: Vastus Lateralis and Medius and Intermedius, TFL: tensor Fascia Lata, Add BLM: adductor (brevis, longus, magnus), Il: Iliacus, Obl: Obliquus (transversus abdominis, internus and externus obliquus), QL: Quadratus Lumborum, VLI: Vastus Lateralis and Intermedius together, VLMI: Vastus Lateralis and Medialis and Intermedius, BF: Biceps Femoris, SMT: Semi Membranosus and Tendinosis, ESM: erector spinae and multifidus, PT: pronator teres, ECRB: Extensor Carpi Radialis Brevis, EPL: Extensor Pollicis Longus, Br: Brachioradialis.
(DOCX)

S5 Table Metrological properties of techniques. (DOCX)

S1 Text Search string. (DOCX)
