# Supplementary material for: Quantifying skeletal muscle volume and shape in humans using MRI: A systematic review of validity and reliability
Source: PLoS One. 2018 Nov 29;13(11):e0207847. doi: 10.1371/journal.pone.0207847 (PMC6264864; doi:10.1371/journal.pone.0207847)
Supplement: S2 Table — (DOCX) [file pone.0207847.s002.docx]

**S2 Table: Quality assessment of the articles included in the review.**

| **Questions** | **1** | **2** | **3** | **4** | **5** | **6** | **7** | **8** | **9** | **10** | **11** | **12** | **13** | **14** | **15** | **subtotal, score/30** | **16** | **17** | **18** | **19** | **20** | **21** | **subtotal score /11** | **total score= Q score/100** |
| --- | --- | --- | --- | --- | --- | --- | --- | --- | --- | --- | --- | --- | --- | --- | --- | --- | --- | --- | --- | --- | --- | --- | --- | --- |
| **Albracht 2008** | 2 | 0 | 1 | 2 | 2 | **2** | 2 | 2 | 0 | 2 | 0 | 2 | 2 | 0 | 1 | 20 | 1 | 2 | 2 | 0 | 0 | 0 | 5 | 61 |
| **Amabile 2016** | 2 | 2 | 2 | 2 | 1 | 1 | 2 | 2 | 0 | 1 | 0 | 2 | 2 | 2 | 2 | 23 | 0 | 2 | 1 | 0 | 0 | 0 | 3 | 63 |
| **Andrew 2015** | 2 | 0 | 0 | 1 | 1 | 1 | 2 | 2 | 2 | 0 | 0 | 1 | 2 | 2 | 2 | 18 | 0 | 2 | 1 | 0 | 0 | 0 | 3 | 51 |
| **Barnouin 2014** | 2 | 2 | 1 | 2 | 2 | 2 | 2 | 2 | 0 | 2 | 0 | 2 | 2 | 2 | 2 | 25 | 2 | 0 | 0 | 2 | 0 | 1 | 5 | 73 |
| **Barnouin 2015** | 2 | 2 | 2 | 2 | 2 | 2 | 2 | 1 | 0 | 2 | 0 | 2 | 2 | 0 | 2 | 23 | 2 | 2 | 2 | 0 | 0 | 0 | 6 | 71 |
| **Belavy 2011** | 1 | 2 | 2 | 0.5 | 2 | 2 | 2 | 2 | 0 | 2 | 0 | 1 | 2 | 2 | 2 | 22 | 2 | 2 | 1 | 0 | 0 | 0 | 5 | 66 |
| **Elliott 1997** | 1 | 0 | 0 | 1 | 0 | 1 | 2 | 2 | 1 | 0 | 0 | 1 | 2 | 1 | 1 | 13 | 0 | 0 | 0 | 1 | 0 | 0 | 1 | 34 |
| **Eng 2007** | 1 | NA | 1 | 0.5 | 2 | 2 | 2 | 2 | 0 | 1 | 0 | 1 | 2 | 1 | 2 | 17 | 2 | 2 | 2 | 1 | 0 | 0 | 7 | 62 |
| **Engstrom 2011** | 2 | 0 | 0 | 1.5 | 2 | 2 | 1 | 2 | 1 | 2 | 0 | 1 | 2 | 0 | 2 | 17 | 0 | 2 | 1 | 0 | 0 | 0 | 3 | 49 |
| **Jolivet 2014** | 1 | 0 | 0 | 1.5 | 2 | 2 | 1 | 2 | 0 | 1 | 0 | 1 | 2 | 2 | 1 | 15 | 0 | 2 | 2 | 0 | 0 | 0 | 4 | 46 |
| **Kim 2017** | 2 | 0 | 0 | 0 | 2 | 1 | 0 | 2 | 1 | 2 | 0 | 1 | 2 | 2 | 2 | 17 | 0 | 2 | 1 | 0 | 0 | 0 | 3 | 49 |
| **Lehtinen 2003** | 2 | NA | 0 | 1,5 | 2 | 2 | 2 | 1 | 1 | 2 | 0 | 2 | 1 | 1 | 2 | 19,5 | 2 | 2 | 1 | 1 | 1 | 0 | 7 | 68 |
| **Le troter 2016** | 2 | 2 | 0 | 2 | 2 | 1 | 2 | 2 | 1 | 1 | 0 | 1 | 2 | 2 | 2 | 22 | 2 | 2 | 2 | 0 | 2 | 0 | 8 | 73 |
| **Lund 2002** | 2 | 2 | 2 | 1.5 | 2 | 2 | 1 | 1 | 0 | 2 | 0 | 2 | 2 | 0 | 2 | 20 | 2 | 2 | 2 | 2 | 2 | 0 | 10 | 73 |
| **Marcon 2015** | 1 | 2 | 2 | 1,5 | 2 | 2 | 2 | 1 | 1 | 2 | 0 | 2 | 2 | 2 | 2 | 24,5 | 1 | 1 | 1 | 0 | 2 | 0 | 5 | 72 |
| **Mersmann 2015** | 2 | 2 | 1 | 2 | 2 | 2 | 2 | 2 | 0 | 2 | 0 | 2 | 2 | 0 | 2 | 23 | 1 | 2 | 2 | 0 | 0 | 0 | 5 | 68 |
| **Mersmann 2014** | 2 | 2 | 0 | 1.5 | 2 | 2 | 2 | 2 | 0 | 2 | 0 | 2 | 2 | 0 | 2 | 20 | 2 | 2 | 2 | 0 | 0 | 0 | 6 | 63 |
| **Moal 2014** | 1 | 0 | 1 | 2 | 2 | 1 | 2 | 2 | 1 | 2 | 0 | 2 | 1 | 0 | 2 | 19 | 0 | 2 | 1 | 2 | 2 | 0 | 7 | 63 |
| **Morse 2007** | 1 | 2 | 1 | 2 | 1 | 2 | 2 | 1 | 0 | 2 | 0 | 1 | 1 | 2 | 1 | 19 | 2 | 2 | 2 | 0 | 0 | 0 | 6 | 61 |
| **Nordez 2009** | 1 | 2 | 1 | 2 | 2 | 1 | 1 | 1 | 0 | 2 | 0 | 2 | 2 | 0 | 2 | 19 | 2 | 2 | 2 | 2 | 2 | 0 | 10 | 71 |
| **Popadic 2011** | 2 | 2 | 1 | 2 | 1 | 1 | 2 | 2 | 0 | 2 | 2 | 2 | 2 | 2 | 1 | 24 | 2 | 2 | 2 | 0 | 0 | 0 | 6 | 73 |
| **Skorupska 2016** | 2 | 2 | 2 | 1.5 | 2 | 2 | 2 | 2 | 2 | 2 | 0 | 2 | 2 | 1 | 1 | 24 | 0 | 0 | 0 | 1 | 0 | 1 | 2 | 63 |
| **Smeulders 2010** | 1 | 2 | 1 | 1.5 | 2 | 1 | 2 | 2 | 0 | 2 | 0 | 2 | 2 | 2 | 2 | 21 | 2 | 0 | 0 | 2 | 2 | 0 | 6 | 66 |
| **Springer 2012** | 1 | 2 | 1 | 2 | 2 | 1 | 2 | 1 | 0 | 2 | 0 | 1 | 2 | 0 | 2 | 19 | 2 | 0 | 0 | 2 | 2 | 0 | 6 | 61 |
| **Sudhoff 2009** | 2 | 2 | 2 | 1 | 2 | 1 | 2 | 1 | 0 | 1 | 0 | 2 | 2 | 2 | 2 | 22 | 0 | 2 | 2 | 2 | 0 | 0 | 6 | 68 |
| **Tingart 2003** | 1 | NA | 1 | 1 | 2 | 1 | 2 | 2 | 0 | 2 | 0 | 1 | 1 | 0 | 2 | 16 | 2 | 2 | 2 | 2 | 2 | 0 | 10 | 63 |
| **Tracy 2003** | 1 | 2 | 0 | 2 | 2 | 2 | 2 | 2 | 0 | 2 | 0 | 2 | 2 | 1 | 2 | 22 | 1 | 2 | 2 | 0 | 0 | 1 | 6 | 68 |
| **Valentin 2015** | 1 | 2 | 2 | 2 | 2 | 1 | 1 | 2 | 2 | 2 | 0 | 2 | 2 | 2 | 1 | 24 | 2 | 0 | 0 | 2 | 0 | 1 | 5 | 71 |
| **Vanmecheln 2017** | 1 | 2 | 2 | 1,5 | 2 | 2 | 2 | 2 | 0 | 1 | 0 | 2 | 2 | 2 | 2 | 23,5 | 2 | 2 | 2 | 0 | 0 | 0 | 6 | 72 |
| **Yamauchi 2017** | 2 | 2 | 2 | 2 | 1 | 2 | 2 | 2 | 1 | 2 | 2 | 2 | 2 | 0 | 2 | 26 | 2 | 2 | 2 | 0 | 0 | 0 | 6 | 78 |

| 1 Aims clearly stated: clear (2), partial (1), no (0) |
| --- |
| 2 Is volunteer/patient consensus obtained before the study?: yes (2), no/not stated (0) |
| 3 Description of the patients/radiographs/ recruitment: clear (2), partial (1), no (0) |
| 4 Description of the population: clear (2), partial (0.5-1.5), no (0) |
| 5 Muscles assessed clearly stated: clear (2), partial (1), no (0) |
| 6 Data acquisition: clear (2), partial (1), no (0) |
| 7 Description of equipment (MRI, software): clear (2), partial (1), no (0) |
| 8 Description of the evaluated muscle volume/shape measure: clear (2), partial (1), no (0) |
| 9 Observer description: experience and specialty (2)/ experience or specialty (1)/ no data (0) |
| 10 Statistical analysis: clear (2), partial (1), no (0) |
| 11 Sample size calculation: yes (2), no (0) |
| 12 Main outcomes of the study clearly stated? clear (2), partial (1), no (0) |
| 13 Key findings supported by the results? yes (2), partial (1), no (0) |
| 14 Description of study limits: yes (2), partial (1), no (0) |
| 15 Key findings answer to the initial objectives: yes (2), partial (1), no (0) |
| 16 Main aim metrological? |
| 17 Concurrent validity evaluation: yes (2), no (0) |
| 18 Description of the gold standard measure: clear (2), partial (1), no (0) |
| 19 Interobserver reliability evaluation: yes (2), without quantification or clinical relevance (1), no (0) |
| 20 Intraobserver reliability: yes (2), without quantification or clinical relevance (1), no (0) |
| 21 Consensus before (guidelines): yes (1), no (0) |
