# Supplementary material for: Quantifying skeletal muscle volume and shape in humans using MRI: A systematic review of validity and reliability
Source: PLoS One. 2018 Nov 29;13(11):e0207847. doi: 10.1371/journal.pone.0207847 (PMC6264864; doi:10.1371/journal.pone.0207847)
Supplement: S3 Table — F: female, M: male, SD: standard deviation, NR: not reported RF: rectus femoris, VI: vastus intermedius, VL: vastus lateralis, VM: vatsus medialis, Qua: quadriceps, Pir: Piriformis, GlMi: Gluteus Minimus, GlMe: Gluteus Medius, GlMa: Gluteus Maximus, FCU: flexor carpi ulnaris, ECU: extensor carpi ulnaris, Sspi: Supraspinatus, Ssca: Subscapularis, Ispi+Tmin: Infraspinatus and Teres minor, ES: Erector Spinae, M: multifidus, RA: rectus abdominis, Ps: Psoas, Sar: Sartorius, Gra: Gracilis, AddM: Adductor Magnus, Add L: Adductor longus, BFL: Biceps Femoris Long head, BFS: Biceps Femoris Short head, ST: Semi Tendinosus, SM: Semi Membranosus, GL: Gastrocnemius Lateralis, GM: Gastrocnemius Medialis, So+FHL: Soleus and flexor hallucis longus, TP: Tibialis Posterior, FDL: flexor digitorum longus, Per LBT: Peroneus (Longus, Brevis, Tertius), TA+EDL+EHL: tibialis anterior and extensor digitorum longus and extensor hallucis longus, So: Soleus, TS: triceps surae, TB: triceps brachii, TA: Tibialis Anterior, VLMI: Vastus Lateralis and Medius and Intermedius, TFL: tensor Fascia Lata, Add BLM: adductor (brevis, longus, magnus), Il: Iliacus, Obl: Obliquus (transversus abdominis, internus and externus obliquus), QL: Quadratus Lumborum, VLI: Vastus Lateralis and Intermedius together, VLMI: Vastus Lateralis and Medialis and Intermedius, BF: Biceps Femoris, SMT: Semi Membranosus and Tendinosis, ESM: erector spinae and multifidus, PT: pronator teres, ECRB: Extensor Carpi Radialis Brevis, EPL: Extensor Pollicis Longus, Br: Brachioradialis FOV: Field of View, NEX: Number of Excitations, TR: Repetition Time, TE: Time to echo. (DOCX) [file pone.0207847.s003.docx]

**S3 Table: Description of the population and MRI technique in the articles included**

|  | **Sample** | | | **Muscles evaluated** | **MRI** | | | |
| --- | --- | --- | --- | --- | --- | --- | --- | --- |
|  | **N of subjects (F, M)** | **type** | **ages (years)** |  | **session** | **Strength and scanner, coil** | **sequence type** | **sequence parameters** |
| **Albracht 2008 [52]** | 13 (13M) | healthy | 29 (SD: 6) | GM, GL, SO | 1 MRI | 0.2T Esaote coil : NR | 3D T1 weighted gradient echo | **TR**: 38 ms **TE**: 16 ms **FOV**: 159*159 mm **flip angle**: NR **NEX**: NR **slice orientation**: axial **slice thickness**: 2mm **slice gap**: 0 mm **resolution**: pixel spacing 0.625*0.625mm |
|  |  |  |  |  |  |  |  |  |
| **Amabile 2016 [53]** | 23 (12F, 11M) | healthy | 19.3 (SD: 0.8) | QL, ES, GlMa, GlMe, GlMi, AddOP, VLI, VM, TFL, RF, Gra, Sar, BFS, BLF, SM, ST, grouped in spine extensors/flexors, hip extensors/flexors, knee extensors/flexors, both sides | 1 MRI | 1.5T GE, coil ; NR | T1, turbo spin echo | **TR**: 427ms **TE**: 11.3ms **FOV**: 16 x 16 x 19.6cm **flip angle**: 160° **NEX**: NR **slice orientation**: axial **slice thickness**: 5mm **slice gap**: 0 mm **resolution**: pixel : 0.82*0.82 mm |
|  |  |  |  |  |  |  |  |  |
| **Andrews 2015 [65]** | - | patients with moderate to severe chronic obstructive pulmonary disease | > 50 | Gra, Sar, BFL, RF, ST, BFS, SM, VI, VM, Add, VL, left side | 1 MRI | 1.5T GE, coil : NR | T1 weighted, fast spin echo | **TR**: 650ms **TE**: 8ms **FOV**: 40cm^3^  **flip angle**: NR **NEX**: NR **slice orientation**: axial **slice thickness**: 5mm **slice gap**: 0 mm **resolution**: pixel : 0.78*0.78 mm |
|  |  |  |  |  |  |  |  |  |
| **Barnouin 2014 [46]** | 20 (11F, 9M) | healthy | 49.7 | RF, VI, VL, VM, Qua, both sides | 1 MRI | 3T Siemens, quadrature birdcage body coil, sets of phased-array receiver coils | 3D 3-point Dixon gradient echo | **TR**: 10msec **TE**: 2.75/3.95/5.15 msec **FOV**: 448*224*320 mm  **flip angle**: 3° **NEX**: 1 **slice orientation:** axial **slice thickness:** 5mm **slice gap**: 3D **resolution:** matrix 448*224 *64 |
|  |  |  |  |  |  |  |  |  |
| **Barnouin 2015 [47]** | 20 (9F, 11M) | healthy | 49,7 | RF, VI, VL, VM, both sides | 1 MRI | 3T Siemens, coil : NR | T1 weighted, 3D 3-point dixon sequence | **TR**: 10 ms **TE**: 2.75/3.95 then 2.75/5.15 ms **FOV**: 448×224×320 mm **flip angle**: NR **NEX**: 1 **slice orientation**: axial **slice thickness**: 5mm **slice gap**: 0mm **resolution:** matrix: 448×224×64 |
|  |  |  |  |  |  |  |  |  |
| **Belavy 2011 [55]** | 20 (20M) | NR | NR | RF, VM, VL, VI, Sar, Gra,Add M, Add L, BFL, BFS,ST, SM, GL, GM, So+FHL, TP, FDL, Per LBT, TA +EDL + EHL, left side | 1st MRI the day of bed rest, then at 2 week intervals (day 14, day 28, day 42 and day 56) through to the end of the bed-rest period | 1.5T Siemens, coil : NR | Proton density turbo spin echo | *thigh, 35 images*  **TR**: 6000msec **TE**: 15msec **FOV**: 480*480mm **flip angle**: 180° **NEX**: NR **slice orientation**: axial **slice thickness**: 10mm **slice gap**: 5mm **resolution**: matrix : 512*512 *lower leg, 30 images* **TR**: 4800msec **TE**: 15msec  **FOV**: 340*340mm **flip angle**: 180° **NEX**: NR **slice orientation**: axial **slice thickness**: 10mm **slice gap**: 5mm **resolution**: matrix : 512*512 |
|  |  |  |  |  |  |  |  |  |
| **Elliot 1997 [66]** | 3 (NR) | NR | 19-50 | GM, GL, So | 1 MRI | 1.5T GE, birdcage extremity coil | T1 weighted, 3D fast gradient echo | **TR**: 100ms **TE**: 10ms **FOV**: 16 x 16 x 19.6cm **flip angle**: 30° **NEX**: NR **slice orientation**: 3D **slice thickness**: 3D **slice gap**: 3D **resolution**: voxel size: 2.73 mm |
|  |  |  |  |  |  |  |  |  |
| **Eng 2007 [54]** | 17 (NR) | cadavers | 82 (SD: 8) | PT (10times ), ECRB (10 times), EPL (10 times), FCU (7 times), BR (6 times) | 1 MRI | 3T GE, coil : NR | T1 weighted, 3D fast spoiled gradient recalled echo pulse sequence | **TR**: 9.2msec **TE**: 3.9msec **FOV**:35 × 35 cm **flip angle**: 30° **NEX**: 1 **slice orientation**: sagittal **slice thickness**: 1.0mm **slice gap**: NR **resolution**: voxel dimension: 1 mm^3^ |
|  |  |  |  |  |  |  |  |  |
| **Engstrom 2011 [67]** | 20 (20M) | healthy (cricket fast bowlers, athletic control subjects) | 18–35 y | QL, Ps, ESM, both sides | 1 MRI | 1.5T Siemens, phased-array spinal coil | T1, turbo spin echo | **TR**: 650ms **TE**: 15ms **FOV**: 250 mm **flip angle**: NR **NEX**: 2 **slice orientation**: axial **slice thickness**: 7mm **slice gap**: 0 mm **resolution**: pixel : 0.98*0.98 mm |
|  |  |  |  |  |  |  |  |  |
| **Jolivet 2014 [68]** | 4 (NR) | NR | 28 (SD: 2.7) | RF, VLMI, Sar, TFL, BFS, BFL, ST, ST, Gra | 1 MRI | 1.5T Philips, Q body coil | T1 spin echo | **TR**: 680ms **TE**: 50ms **FOV**: NR **flip angle**: NR **NEX**: NR **slice orientation**: axial **slice thickness**: 10 mm **slice gap**: NR **resolution**: 0.78*0.78 mm |
|  |  |  |  |  |  |  |  |  |
| **Kim 2017 [29]** | 5 (-'-) | NR | NR | Sspi | 1 MRI | 3T, coil : NR | 3D | **TR**: NR **TE**: NR **FOV**: NR **flip angle**: NR **NEX**: NR **slice orientation**: sagittal **slice thickness**: 0.7mm **slice gap**: 3D **resolution**: pixel : 0.63*0.63mm |
|  |  |  |  |  |  |  |  |  |
| **Lehtinen 2003 [56]** | 10 (6F, 4M) | cadavers | 76 (67-82) | Sspi,Ssca, Ispi+Tmin | 1 MRI | 1.5T GE, shoulder coil | T1 weighted gradient echo | **TR**: 100 ms **TE**: minimum **FOV**: 260*260 mm **flip angle**: NR **NEX**: 1 **slice orientation**: coronal **slice thickness**: 5mm **slice gap**: 5mm **resolution:** matrix: 256*128 |
|  |  |  |  |  |  |  |  |  |
| **Le Troter 2016 [48]** | 7 (7M) | healthy | 32 (SD: 7) | RF, VI, VM, VL, Qua, right side | 2 MRI sessions repeated twice | 1.5T Siemens, flexible surface 6-channel body coil | T1-weighted, gradient echo | **TR**: 549 ms **TE**: 13 ms **FOV**: 220*220 mm2 **flip angle**: NR, **NEX**: NR, **slice orientation**: axial, **slice thickness**: 6mm, **slice gap**: 6mm, **resolution**: matrix 576 × 576 |
|  |  |  |  |  |  |  |  |  |
| **Lund 2002 [49]** | 11 (4F, 7 M) | healthy | 24–40 | TA+EDL+EHL, left side | 1 MRI | 1.5T Philips, knee coil | T2 weighted fast-field echo fat-saturated (spectral inversion recovery, SPIR) | **TR**: 56 ms **TE**: 14 ms **FOV**:180 *180 mm **flip angle**:15° **NEX**: NR **slice orientation**: axial **slice thickness**: 1.5 mm **slice gap:** 0mm **resolution:** matrix: 256*256 |
|  |  |  |  |  |  |  |  |  |
| **Marcon 2015 [9]** | 34 (12F, 22M) | persons with ACL reconstructions | F: 31.3 (SD: 8.8) M: 30.9 (SD: 6.5) | Qua | 1 MRI | 1.5T GE, coil : NR | T1 weighted, 3D spoiled dual gradient-echo water signal only | **TR**: 6.14 ms **TE**: 2.1/4.2 ms **FOV**: NR **flip angle**: 5° **NEX**: 2 **slice orientation**: axial **slice thickness**: 6mm **slice gap**: 3D **resolution:** matrix: 320*224 |
|  |  |  |  |  |  |  |  |  |
| **Mersmann 2014 [57]** | 21 (NR) | healthy | 25 (SD: 8) | GM, GL, SO, TS, right side | 1 MRI | 1.5 T Siemens, coil : NR | T1, gradient echo | **TR**: 3.11 ms **TE**: 1.18 ms **FOV**: 244*449 mm **flip angle**: NR **NEX**: NR **slice orientation**: axial **slice thickness**: 1.8mm **slice gap**: 0mm **resolution**: NR |
|  |  |  |  |  |  |  |  |  |
| **Mersmann 2015 [58]** | 37 (20F, 17M) | healthy | W: 31 (SD: 17) M: 32 (SD: 16) | VL, VM, VI, one side | 1 MRI | 1.5T Siemens, coil : NR | T1 weighted, turbo spin echo | **TR**: 641 ms **TE**: 11 ms **FOV**: 230*420 mm **flip angle**: NR **NEX**: NR **slice orientation**: axial **slice thickness**: 4mm **slice gap**: 0.8mm **resolution**: NR |
|  |  |  |  |  |  |  |  |  |
| **Moal 2014 [59]** | 2 (2F) | healthy | 35, 38 | Add BLM, BF, ES, GlMa, GlMe, GlMi, Gra, Il, Obl, Ps, QL, RA, RF, Sar, SMT, TFL, VLI, VM | 1 MRI, 2 sequences | 3T Siemens, 24-channel spine matrix coil and three 4-channel flex coils | T1weighted turbo spin echo, T1 weighted turbo spin echo for the 3 point Dixon method | *T1weighted TSE*  **TR**: 1220 ms **TE**: 11 ms **FOV**: NR **flip angle**: 150° **NEX**: NR **slice orientation**: axial **slice thickness**: 5mm **slice gap**: 5mm **resolution**: 0.98*0.98mm, *T1 weighted TSE for the 3 point Dixon method*  **TR**: 829 ms **TE**: 15.7 ms **FOV**: NR **flip angle**: 150° **NEX**: NR **slice orientation**: axial **slice thickness**: 5mm **slice gap**: 5mm **resolution**: pixel : 0.98*0.98mm |
|  |  |  |  |  |  |  |  |  |
| **Morse 2007 [60]** | 18 (18M) | healthy | 23.9 (SD: 3.4) | Qua, VL, VM, VI, RF, right side | 1 MRI | 0.2T Esaote coil : NR | T1 weighted, gradient echo | **TR**: 100 ms **TE**: 16 ms **FOV**: 330*254 mm **flip angle**: NR **NEX**: NR **slice orientation**:11 axial planes **slice thickness**: 5mm **slice gap**: NR **resolution**: matrix 256 * 256 11 slices along the femur |
|  |  |  |  |  |  |  |  |  |
| **Nordez 2009 [27]** | 10 (10M) | healthy | 29 (SD: 4) | Qua (VL+VI+VM+RF) | 1 MRI | 1.5T Siemens, angiography radiofrequency coil | volume interpolated GRE T1 | **TR**: 4.47 ms **TE**: 2.10 ms **FOV**: 400*400 mm flip angle: NR **NEX**: NR **slice orientation**: axial **slice thickness**: 4mm **slice gap**: 0mm **resolution**: voxel: 0.78 *0.78*4 mm |
|  |  |  |  |  |  |  |  |  |
| **Popadic 2011 [50]** | 35 (35M) | healthy | 21.6 (SD: 2.5) | TB, both sides | 2 MRIs 1 before/ 1 after training | 1.5T Siemens extremity coil | T1 weighted, gradient echo | **TR**: 232msec **TE**: 4.76msec **FOV**: 162*288 mm **flip angle**: NR **NEX**: NR **slice orientation**: axial **slice thickness**: 10mm **slice gap**: 3mm **resolution**: matrix 288*512 |
|  |  |  |  |  |  |  |  |  |
| **Skorupska 2016 [61]** | 100 (63F, 37M) | 71 low back pain, 29 healthy | low back pain 47.7 (SD: 8.4); healthy 47.6 (SD: 9.9) | Pir, GlMi, GlMe, GlMa, both sides | 1 MRI | 1.5T GE, coil : NR | T2 weighted | **TR**: 3500 ms **TE**: 110 ms **FOV**: 320*324 mm **flip angle**: NR **NEX**: NR **slice orientation**: sagittal **slice thickness**: 4mm **slice gap**: 0mm **resolution**: matrix 320*224 |
|  |  |  |  |  |  |  |  |  |
| **Smeulders 2010 [62]** | 10 (5F, 5M) | healthy | 30 (20-35) | FCU, ECU, right side | 2 MRIs, 1 week between sessions | 3T Philips, flexibles surface coils with two elliptical elements | T1 weighted axial spin echo | **TR:** 500 ms **TE**: 12 ms **FOV**: 160*160 mm **flip angle**: NR **NEX**: NR **slice orientation**: axial **slice thickness**: 6mm **slice gap**: NR **resolution**: matrix : 300*300 |
|  |  |  |  |  |  |  |  |  |
| **Springer 2012 [63]** | 10 (6F, 4M) | with unilateral total hip arthroplasty | 61.8 (SD: 12.2; 35–76) | GlMe, GlMi, OE, both sides | 1 MRI, 12 months after hip arthroplasty | 1.5T Siemens, flexible wraparound phased-aray surface coil | T1 weighted turbo spin echo | *OE*  **TR**: 667 ms  **TE**: 12 ms **FOV**: 400*400 mm **flip angle**: 150° **NEX**: NR **slice orientation**: coronal **slice thickness**: 5mm **slice gap**: NR **resolution**: matrix 512*256 *Glutei muscles*  **TR**: 667 ms **TE**: 12 ms **FOV**: 420*275.52 mm **flip angle**: NR **NEX**: NR **slice orientation**: axial **slice thickness**: 6mm **slice gap**: NR **resolution**: matrix 512*168 |
|  |  |  |  |  |  |  |  |  |
| **Sudhoff 2009 [64]** | 10 (10M) | healthy | 29 (SD: 4) | SM, ST, BFS, BFL, Sar, TFL, Gra, VLI, VM, RF, GM, GL | 1 MRI | 1.5T Siemens, , angiography radiofrequency coil | volume interpolated GRE T1 | **TR**: NR **TE**: NR **FOV**: NR **flip angle**: NR **NEX**: NR **slice orientation**: axial **slice thickness**: 4 mm **slice gap**: 0 mm **resolution**: pixel : 0.78*0.78 mm |
|  |  |  |  |  |  |  |  |  |
| **Tingart 2003 [25]** | 10 (6F, 4M) | cadavers | 76 (67-82) | Sspi,Ssca, Ispi+Tmin | 1 MRI | 1.5T GE, linear shoulder array coil | T1 weighted fast spin echo | **TR**: 650msec **TE**: 10msec **FOV**: 180*180 mm **flip angle**: NR **NEX**: NR **slice orientation**: oblique sagittal **slice thickness**: 3mm **slice gap**: 0mm **resolution**: matrix 512*224 |
|  |  |  |  |  |  |  |  |  |
| **Tracy 2003 [26]** | 47 (21F, 26 M) | healthy | young: 26 (SD: 3) (N= 23); older: 69 (SD: 3) (N= 24) | Qua, trained side | 2 MRIs 1 before/ 1 after training | 1.5T Picker Edge, coil : NR | T1 weighted | **TR**: 700 ms **TE**: 14 ms **FOV**: 50cm **flip angle**: NR **NEX**: NR **slice orientation**: axial **slice thickness**: 9mm **slice gap**: 1mm **resolution**: matrix 256*256 |
|  |  |  |  |  |  |  |  |  |
| **Valentin 2015 [45]** | 10 (10M) | healthy | 5 young (18-25) 5 mature (45-60) | ES, M, RA, Ps both sides | 1 MRI | 1.5T Siemens, **coil** : NR | T1 weighted gradient echo | **TR**: 9.3 ms **TE**: 4.6 ms **FOV**: rectangular, 78% **flip angle**: NR **NEX**: NR **slice orientation**: axial **slice thickness**: 10mm **slice gap**: NR **resolution**: NR |
|  |  |  |  |  |  |  |  |  |
| **Vanmechelen 2017 [51]** | 44 (11F, 33M) | 21 bilateral cerebral palsy, 23 healthy | cerebral palsy: 14.7 (SD: 3) helathy: 16.8 (SD: 3.3) | GM, SOL, TA, RF, SM, ST, left side | 1 MRI | 1.5T Philips (22 subjects) 3T Philips (22 subjects) , quadrature body coil | T2 weighted, 3 point Dixon | *For 1,5T scanner*  **TR:** 4.6ms  **TE:** 13ms **FOV:** NR **flip angle:** 20°  **slice orientation:** axial  **slice thickness:** 5mm  **slice gap:** NR  **resolution:** voxel size 0.9*0.9*0.5mm *For 3T scanner*  **TR:** 2.11ms  **TE:** 5.2ms  **FOV:** NR  **flip angle:** 10°  **slice orientation:** axial **slice thickness:** 5mm  **slice gap:** NR  **resolution:** voxel size 0.9*0.9mm |
|  |  |  |  |  |  |  |  |  |
| **Yamauchi 2017 [28]** | 24 (12F, 12M) | 12 with knee osteoarthritis grade 1 and 12 with knee osteoarthritis grade >=2 | 74.3 (SD: 4.4) | VL, VM, VI, RF, SM, ST, BFS, BFL, painful side | 1 MRI | 1.5T Toshiba | T1 weighted, turbo spin-echo coil: NR | **TR**: 625ms **TE**: 15ms **FOV**: 250mm **flip angle**: NR **NEX**: NR **slice orientation**: axial **slice thickness**: 10mm **slice gap**: 0mm **resolution**: matrix: 512*512 |

F: female, M: male, SD: standard deviation, NR: not reported

RF: rectus femoris, VI: vastus intermedius, VL: vastus lateralis, VM : vatsus medialis, Qua : quadriceps, Pir : Piriformis, GlMi : Gluteus Minimus, GlMe : Gluteus Medius, GlMa : Gluteus Maximus, FCU: flexor carpi ulnaris, ECU: extensor carpi ulnaris, Sspi: Supraspinatus, Ssca: Subscapularis, Ispi+Tmin: Infraspinatus and Teres minor, ES: Erector Spinae, M: multifidus, RA: rectus abdominis, Ps: Psoas, Sar: Sartorius, Gra: Gracilis, AddM: Adductor Magnus, Add L: Adductor longus, BFL: Biceps Femoris Long head, BFS: Biceps Femoris Short head, ST: Semi Tendinosus, SM: Semi Membranosus, GL: Gastrocnemius Lateralis, GM: Gastrocnemius Medialis, So+FHL: Soleus and flexor hallucis longus, TP: Tibialis Posterior, FDL: flexor digitorum longus, Per LBT: Peroneus (Longus, Brevis, Tertius), TA+EDL+EHL: tibialis anterior and extensor digitorum longus and extensor hallucis longus, So: Soleus, TS: triceps surae, TB: triceps brachii, TA: Tibialis Anterior, VLMI: Vastus Lateralis and Medius and Intermedius, TFL: tensor Fascia Lata, Add BLM: adductor (brevis, longus, magnus), Il: Iliacus , Obl: Obliquus (transversus abdominis, internus and externus obliquus), QL: Quadratus Lumborum, VLI: Vastus Lateralis and Intermedius together, VLMI: Vastus Lateralis and Medialis and Intermedius, BF: Biceps Femoris, SMT: Semi Membranosus and Tendinosis, ESM : erector spinae and multifidus, PT: pronator teres, ECRB : Extensor Carpi Radialis Brevis, EPL : Extensor Pollicis Longus, Br : Brachioradialis

FOV: Field of View, NEX: Number of Excitations, TR: Repetition Time, TE: Time to echo
