# Supplementary material for: Quantifying skeletal muscle volume and shape in humans using MRI: A systematic review of validity and reliability
Source: PLoS One. 2018 Nov 29;13(11):e0207847. doi: 10.1371/journal.pone.0207847 (PMC6264864; doi:10.1371/journal.pone.0207847)
Supplement: S1 Text — (DOCX) [file pone.0207847.s006.docx]

**Pubmed – Medline Search String, December 26, 2017:**

(((((((((((MRI*[Text Word]) OR magnetic resonance imaging[MeSH Terms])) AND (((("muscles"[MeSH Terms]) OR "muscle, skeletal"[MeSH Terms])) OR ((muscle*[Text Word]) OR muscul*[Text Word])))) AND (((("models, anatomic"[MeSH Terms]) OR "organ size"[MeSH Terms])) OR ((((((volum*[Text Word]) OR cross sectional area[Text Word]) OR three-dimension*[Text Word]) OR 3D[Text Word]) OR shape[Text Word]) OR segmentation[Text Word])))) AND ((((("reproducibility of results"[MeSH Terms]) OR "data accuracy"[MeSH Terms])) OR (((((((reliability[Text Word]) OR reproducibility[Text Word]) OR validity[Text Word]) OR repeatability[Text Word]) OR accuracy[Text Word]) OR measur*[Text Word]) OR metrologic*[Text Word])) OR "validation studies"[Publication Type]))) NOT myocard*[Text Word]) NOT cardia*[Text Word]) NOT cardiol*[Text Word]

**Cochrane Library search string, December 30, 2017**

(("MRI" OR "magnetic resonance imaging") AND ("muscle" OR "skeletal muscle" OR "muscul*" ) AND ("volum*" OR "cross sectional area" OR "three dimension*" OR "3D" OR "shape" OR "segmentation" OR "organ size") AND ("reliability" OR "reproducibility" OR "repeatability" OR "validity" OR "accuracy" OR "measur*" OR "metrologic*" OR "validation stud*")) in all Studies

**Web of Science Search string, December 29, 2017**

(TS = (((“MRI” or “magnetic resonance imaging”) AND (“muscle” OR “skeletal muscle” OR “muscul*”) AND (“volum*” OR “cross sectional area” OR “three-dimension*” OR “3D” OR “shape” OR “segmentation” OR “organ size”) AND ("reliability" OR "reproducibility" OR "repeatability" OR "validity" OR "accuracy" OR "measur*" OR "metrologic*" OR "validation stud*")) NOT (“cardiol*” or “cardia*”)) *AND* **DOCUMENT TYPES:** (Article)

**Scopus search string, January 2, 2018**

( TITLE-ABS-KEY ( ( "MRI" OR “magnetic resonance imaging” ) AND ( "muscle" OR "skeletal muscle" OR "muscul*" ) AND (“volum*” OR “cross sectional area” OR “three-dimension*” OR “3D” OR “shape” OR “segmentation” OR “organ size”) AND ( "reliability" OR "reproducibility" OR "repeatability" OR "validity" OR “accuracy” OR “measur*” OR “metrologic*” OR “validation stud*) ) ) AND NOT TITLE-ABS-KEY ( "cardiol*" OR “cardia*” ) ) AND  ( LIMIT-TO ( SRCTYPE ,  *"j"* ) )
